# Cow Longevity and Reasons and Risk Factors for Culling in South African Holstein and Jersey Dairy Herds

**DOI:** 10.3390/ani15203012

**Published:** 2025-10-17

**Authors:** Lerato Matjila, Khathutshelo Nephawe, Yandisiwe Sanarana, Bekezela Dube, Cuthbert Banga

**Affiliations:** 1 Department of Animal Sciences, Faculty of Science, Tshwane University of Technology, Private Bag X680, Pretoria 0001, South Africa; nephaweka@tut.ac.za (K.N.); cbanga@buan.ac.bw (C.B.); 2 ARC-Animal Production, Private Bag X2, Irene 0062, South Africa; sanaranay@gmail.com (Y.S.); dubeb@arc.agric.za (B.D.); 3 Department of Animal Sciences, Faculty of Animal and Veterinary Sciences, Botswana University of Agriculture and Natural Resources, Private Bag, Gaborone 0027, Botswana

**Keywords:** involuntary culling, productive lifespan, herd management, profitability, sustainability

## Abstract

This study investigated cow longevity, culling reasons, and risk factors influencing culling in South African Holstein and Jersey dairy herds. Lactation records of 1,150,625 Jersey and 1,534,875 Holstein cows from 1864 herds, recorded through the National Milk Recording Scheme during the period 2000 to 2019, were analyzed. Longevity was calculated as length of productive life and number of completed lactations. Logistic binary regression was conducted to estimate the odds ratios (OR) for culling among different calving seasons, parities, and herd sizes. Holstein cows had mean productive life of 739.33 ± 434.31 days and 2.37 ± 1.08 lactations, while Jersey cows averaged 696.81 ± 415.44 days productive life and 2.47 ± 1.13 lactations. Leading reasons for culling were infertility (37.94 ± 0.48% Holstein; 30.46 ± 0.63% Jersey), mastitis (18.15 ± 0.38% Holstein and 18.16 ± 0.53% Jersey), and low milk yield (11.76 ± 0.32% Holstein and 19.76 ± 0.55% Jersey). Summer calving, third parity, and small herd size had the highest odds of culling. These findings suggest that herd management practices and selection objectives in South Africa should place high emphasis on cow fertility and udder health. Furthermore, cows calving in summer and those in third parity or small herds require particular attention to minimize culling. Such measures may help to reduce involuntary culling rates and thus increase herd profitability as well as dairy industry sustainability.

## Simple Summary

Knowledge of culling reasons and the associated risk factors, in dairy herds, is essential for assisting farmers to develop management strategies to minimize economic losses due to involuntary culling. Such knowledge is, however, limited for dairy cattle performing in South Africa. This study examined culling records of Holstein and Jersey cows from 1864 South African herds with complete records from the period of 2000 to 2019. Average productive life of cows was between 2 and 3 lactations. Infertility, mastitis and low milk yield were identified as the most common culling reasons for both Holstein and Jersey cows. Calving season, parity, and herd size significantly influenced the risk of culling, with cows calving in summer, and those in third parity or small herds having the highest risk. Collectively, these culling reasons and risk factors have an impact on cow longevity, herd productivity, and profitability. Strategic herd management practices can be developed based on these findings, with increased focus on improving fitness and functional traits, to enhance cow resilience and ensure profitable and sustainable dairy farming.

## 1. Introduction

The demand for milk and dairy products is steadily increasing globally with projections indicating that approximately 1.2 billion additional consumers will require these products by 2030 [1]. This growing demand underscores the vital role of dairy farming in meeting the nutritional requirements of a rising population, as well as contributing significantly to food security and global economic growth. It also presents an opportunity for farmers to increase milk production; however, there is a need to implement strategic herd management practices to enhance production efficiency and ensure the sustainability of dairy farming.

Culling is a critical herd management practice that entails voluntary or involuntary removal of cows from the herd for various reasons. Farmers often cull cows to manage herd size, improve performance, reduce economic losses, and enhance profitability [2,3]. In dairy herds, culling rates are typically used as a measure of herd performance. The optimum culling rate has been reported to range between 25% and 32% annually [4,5]. Higher culling rates may indicate underlying management or health issues, while lower rates often reflect good herd performance. Nevertheless, low culling rates may at times not indicate effective herd management practices, simply because they may likely result from retaining unproductive and diseased cows such as those with mastitis or lameness [6,7], subsequently lowering herd performance and increasing the risk of disease transmission and veterinary costs.

High culling rates cause increased heifer replacement costs [6,8] and reduced herd milk production, resulting in decreased herd profitability [9,10]. Significant financial losses are incurred when cows are culled prematurely, before reaching their maximum milk production level. Excessive culling may also indicate poor animal welfare status or farm management challenges [11]. Conversely, minimal culling is generally associated with increased cow longevity, meaning lower replacement costs and a higher proportion of mature, high-yielding cows within the herd, which improves profitability [12]. Previous research has shown that cow longevity has declined in South African dairy herds, particularly in Gauteng, Eastern Cape, and KwaZulu-Natal over the past two decades [13]. This trend is consistent with global patterns, where a reduction in productive lifespan by 0.9 to 3.04 years was recorded between 1967 and 2019 in several leading milk-producing countries [14].

Recent trends indicate an increase in involuntary culling in dairy herds, mainly due to reasons such as diseases and reproductive problems [15]. Higher rates of involuntary culling do not only increase herd management costs [16] but also limit the opportunity for voluntary replacements, such as selection for increased production, which adversely affects genetic progress and long-term herd development [17]. Effective management of culling rates is, therefore, critical to improving profitability and achieving sustainability of dairy farming [2]. Knowledge of the specific reasons and risk factors associated with culling is essential as it will assist farmers to develop appropriately targeted management strategies to reduce involuntary culling. Although reasons and risk factors for culling have been identified in many dairy cattle populations [2,6,18], there has been limited research on culling in South African dairy herds. Factors such as environmental conditions, management systems, farm size, and morbidity of herds, which may influence culling risk, vary from one country and dairy cattle population to another [4,8,19]. South African dairy herds are distinct from global dairy cattle populations based on the farmers management practices that are influenced by harsh climatic conditions, including droughts, high temperatures, and irregular rainfall [20]. High cost of feed, electricity and veterinary services [21], as well as lack of appropriate biosecurity measures resulting in disease outbreaks pose challenges to the herds [22].

A recent study undertaken in Eastern Cape, South Africa, examined causes of culling and mortality in dairy herds, leaving a large gap in understanding of the situation at national level [13]. Lack of such critical knowledge is a drawback to the achievement of improved profitability and long-term sustainability of the South African dairy industry. Thus, the objective of this study was to explore the reasons and risk factors for culling and their impact on longevity of Holstein and Jersey cows in South African dairy herds. This knowledge can assist farmers to control culling rates more effectively, lower costs resulting from involuntary culling, and achieve higher rates of genetic improvement, thereby increasing herd profitability.

## 2. Materials and Methods

### 2.1. Study Population and Data Collection

The study was based on lactation records of cows in Holstein and Jersey herds participating in the national milk recording scheme from the period of 2000 to 2021. The data were extracted from the Integrated Registration and Genetic Information System (INTERGIS), which is the national livestock database of South Africa (SA) managed by the Agricultural Research Council (ARC). Field technicians of the ARC collect performance data recording during regular 4–6 week interval visits to participating herds. Holstein and Jersey were used for the study as they are the most dominant dairy cattle breeds in South Africa, comprising over 90% of the national dairy cattle population [23]. The average herd size was 338 cows for Holstein and 403 for the Jersey. The management systems utilized on the farms include extensive pasture grazing, intensive housing with total mixed ration (TMR) feeding, and a combination of the two. Milking systems varied, with some herds using conventional techniques and others utilizing modern automated systems, typically milking cows two to three times per day. Artificial insemination was the primary mating method, and calving was either seasonal or year-round.

### 2.2. Data Preparation and Editing

Data comprised cow lactation records, consisting of details such as breed, herd code, cow identification number, birth date, lactation number, calving date, lactation end date, and lactation termination reason. For those cows that were culled at the end of the lactation, the disposal reason was specified by means of a code. Culling reasons recorded included infertility, udder problems, feet and leg problems, mastitis, injury, low milk yield, and health issues. Udder problems included structural defects such as pendulous udder, uneven udder attachment, poorly placed teats, udder swelling, and teat injuries. Feet and leg problems referred to conditions such as weak pasterns, straight hocks, and cow hocked legs, along with joint swelling, injuries, and signs of lameness. All infectious diseases, metabolic disorders and parasitic diseases were classified as health issues. The initial data collected during the period from 2000 to 2021 across the country was used. It included 4,328,240 lactation records of 2,252,580 cows from 3465 dairy herds. These records were subsequently edited and updated in the Statistical Analysis System software (SAS, version 9.4, 2012). A structured data cleaning process excluded herds with fewer than 20 cows per herd-year or records missing key information such as birth date, calving date, lactation number, or culling reason. Cows without a first lactation record were also removed. Herds that discontinued milk recording at some point were not included as they contained censored records. Cow longevity was expressed as number of days of productive life (i.e., first calving to disposal) and number of completed lactations, where a lactation was considered complete if the cow was in milk for at least 120 days. For each lactation, the calving age was restricted based on criteria shown in Table 1, to remove outliers, following procedures used for the national genetic evaluation system [24].

**Figure 1:**
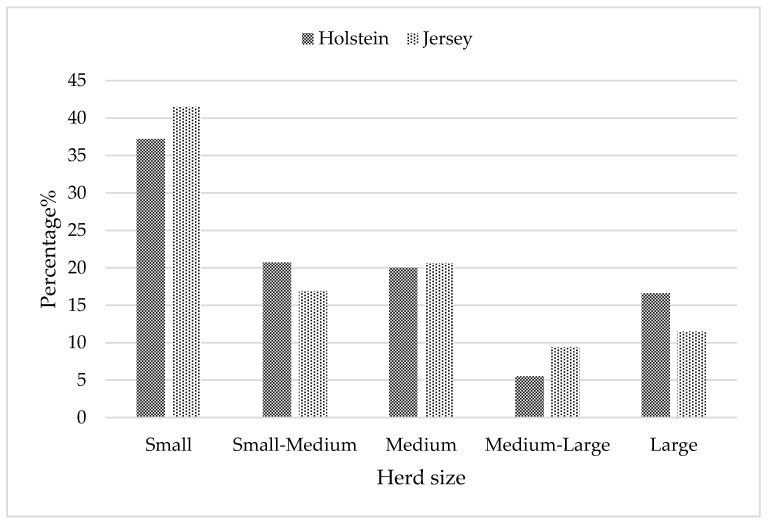
Frequency distribution of Holstein and Jersey herds based on average annual herd size.

Calving season was defined as summer (December–February), autumn (March–May), winter (June–August), and spring (September–November). Herd size was categorized into five groups based on average annual herd size: small (<50 cows), small to medium (51–100 cows), medium (101–200 cows), medium to large (201–300 cows), and large (>300 cows) in Figure 1. Parity was classified into four groups: 1, 2, 3, and 4. After editing, the final data set with complete records over 19 years (2000 to 2019) was used for the downstream analysis. This consisted of 1534,875 lactation records of 663 800 Holstein cows from 987 herds, and 1,150,625 lactation records of 595,097 Jersey cows from 877 herds.

### 2.3. Statistical Analysis

Statistical analyses were conducted separately for each breed, using the Statistical Analysis System software (SAS, version 9.4, 2012). Descriptive statistics for length of productive life (days) and number of completed lactations were computed by the PROC MEANS procedure, whereas the PROC FREQ procedure was applied to obtain the proportion of cows surviving to a specified length of productive life. Frequency distribution of culling reasons, and proportions of voluntary and involuntary culling, were also determined using the PROC FREQ procedure. Binary logistic regression was conducted using the PROC LOGISTIC procedure to identify factors influencing the likelihood of culling and estimate odds ratios (OR) of culling for the groups within each factor, which was significant (*p* < 0.05). The logistic model fitted culling with two possible outcomes (1 = culled, 0 = not culled) based on the survival of cows up to the fourth lactation. The following general model (model 1) was fitted:

Logit (p) = β_0_ + β_1_X_1_ + β_2_X_2_ + β_3_X_3+_ + Ɛ (1)

where

Logit (p) is the probability of culling (1 = yes, 0 = no);
Β_0_ is the intercept of the regression line;
β_1_ is the regression coefficient for parity;
X_1_ is parity (discrete predictor);
β_2_ is the regression coefficient for herd size;
X_2_ is herd size (discrete predictor);
β_3_ is the regression coefficient for calving season;
X_3_ is calving season (discrete predictor);
Ɛ is the random error.

## 3. Results

### 3.1. Descriptive Statistics for Cow Longevity

Table 2 indicates that the average length of productive life was 739.33 ± 434.31 and 696.81 ± 415.44 days, and the corresponding average number of completed lactations was 2.37 ± 1.08 and 2.47 ± 1.13 for Holstein and Jersey cows, respectively.

### 3.2. Cow Survival Rate by Length of Productive Life

**Figure 2:**
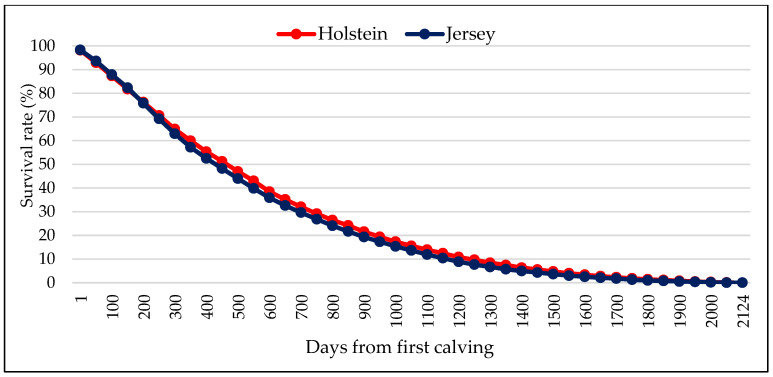
Survival rate of Holstein and Jersey cows by length of productive life in South African dairy herds between 2000 and 2019.

Survival of cows to a given day of productive life is illustrated in Figure 2. Less than 40% of cows of both breeds survived to 600–700 days after their first calving, indicating large-scale culling in the first two lactations. Rate of survival declined more gradually after 600 days of productive life, with 17% and 15% of Holstein and Jersey cows, respectively, reaching 1000 days.

### 3.3. Cow Survival Rate Based on Different Parities

**Figure 3:**
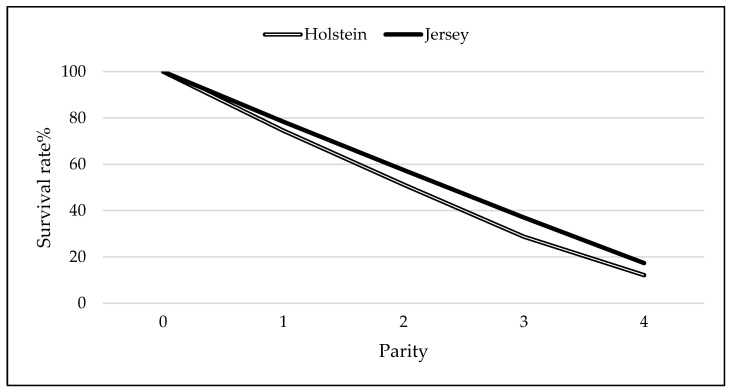
Survival rate of Holstein and Jersey per parity from South African dairy herds between 2000 and 2019.

Survival rate of Holstein and Jersey per parity are presented in Figure 3. On the first parity, the survival rates were 74.52% and 78.37% Holstein and Jersey cows, respectively. During the second parity, only half of Holstein (51.22%) and Jersey (57.53%) cows survived, showing a sharp decline in early parities. The rate of survival continued to decrease gradually as parities advanced, with 28.73% and 37.02% of Holstein and Jersey cows reaching the third parity. By the fourth parity, survival rates had decreased to 12.13% and 17.34% for Holstein and Jersey cows, respectively.

### 3.4. Distribution of Culling Reasons

**Figure 4:**
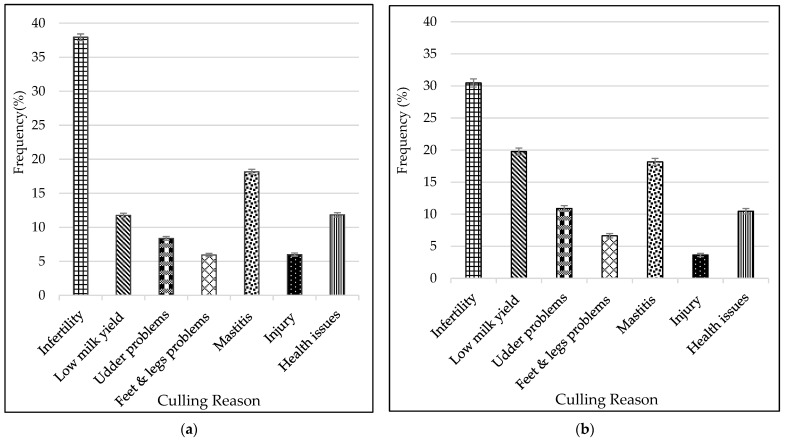
Culling reasons frequency distributions for the Holstein (**a**) and Jersey (**b**) cows based on records collected from 2000 to 2019 in South African dairy herds.

Distribution of reasons for disposal of cows from the studied herds are displayed in Figure 4a,b for Holstein and Jersey, respectively. Infertility was by far the most prevalent reason for culling, accounting for 37.94 ± 0.48% and 30.46 ± 0.63% of the total number of Holstein and Jersey cows culled, respectively. Mastitis was the second common reason for culling Holstein cows, with a prevalence of 18.15 ± 0.48%, followed by health-related issues at 11.84 ± 0.32% and low milk yield at 11.76 ± 0.32%. For the Jersey, low milk yield (11.76 ± 0.55%) was the second leading reason for culling, closely followed mastitis (18.16 ± 0.53%) and udder problems (10.89 ± 0.44%). The least common reasons for culling were feet and leg problems for Holsteins (5.94 ± 0.23%) and injuries for Jerseys (3.64 ± 0.26%). Holstein cows were also culled for udder problems (8.47 ± 0.27%) and injury (5.99 ± 0.23%), while reasons for culling Jersey cows included health issues (10.46 ± 0.42%) and feet and legs problems (6.63 ± 0.34%).

### 3.5. Proportions of Involuntary and Voluntary Culling

**Figure 5:**
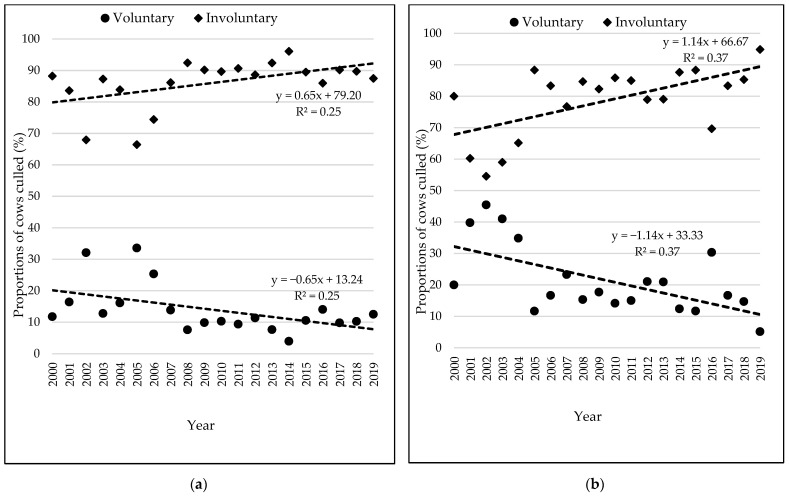
Annual trends in the proportions of voluntary and involuntary culling for Holstein (**a**) and Jersey (**b**) cows based records collected from 2000 to 2019 in South African dairy herds.

Trends in annual proportions of involuntary and voluntary culling during the study period (2000–2019) are shown in Figure 5. Cows were mainly removed from the herds involuntarily, with the proportion of involuntary culling ranging from 66.43% to 96.09% for Holsteins and 54.55% to 94.87% for Jerseys. Voluntary culling peaked in 2005 for the Holstein (33.57%) and in 2002 for Jersey (45.45%). Overall, there was a steady decline in the proportion of cows culled voluntarily during the study period, reaching below 20% for Holstein and less than 10% for Jersey by 2019. Conversely, involuntary culling increased gradually from 2006 (74.43%) to 2019 (87.5%) in Holstein and 2003 (58.99%) to 2019 (94.87%) in Jersey.

### 3.6. Risk Factors for Culling

Table 3 presents the results of the test to assess factors influencing the risk of culling in the studied populations. The likelihood of cows being removed from the herd was significantly influenced by calving season (*p* < 0.0001) and parity and herd size (*p* < 0.05). The estimated odds ratios for culling among the groups within each of these factors are presented in Table 4 for both Holstein and Jersey.

### 3.7. Calving Season

Summer calving had a higher odds ratio for culling than the rest of the seasons in both Holstein and Jersey cows. Holstein cows that calved during winter, autumn and spring had, respectively, 24.8%, 17.1%, and 15% lower odds of being culled compared to cows that calved in summer. The corresponding estimates for the Jersey were 38.3%, 15.3%, and 12.1%, respectively. These differences were, however, only significant between summer and winter (*p* < 0.05).

### 3.8. Parity

Third parity cows had the highest odds for culling, which was 46.1% and 46.5% more than for those in first lactation, for Holstein and Jersey, respectively. This was followed by second lactation Holstein (20.5%) and Jersey (17%) cows. Fourth parity was associated with the lowest risk of culling, which was estimated at 14.5% and 10.3% lower than for first parity in Holstein and Jersey, respectively. No significant difference (*p* > 0.05) was, however, detected between first and second parity Jersey cows.

### 3.9. Herd Size

The highest odds ratio for culling was estimated for small herds in both Holstein and Jersey cows. For Holstein, cows in medium–large herds had the lowest odds for culling at 51.3% while for Jersey cows it was observed in medium-sized herds at 55.4%. The difference between small and medium–large herds was, however, not significant (*p* > 0.05) for Jersey.

## 4. Discussion

Culling in dairy herds is essential for sustaining productivity and enhancing profitability. This is achieved by replacing unproductive cows and removing those with contagious diseases, resulting in improved health status of the herd, genetics, and optimized use of resources. An effective culling strategy seeks to minimize involuntary culling, maximize cow longevity, and promote optimal rates of voluntary culling. Attainment of these goals is, however, dependent on careful, strategic, and well-informed culling of cows from the herd. Thus, knowledge about aspects such as the major causes for removal of cows from the herd and their related risk factors is critical for ensuring proper management of culling. Several studies have investigated the reasons and contributing factors for culling in dairy cattle populations worldwide [2,6,19]. However, reasons for culling cows and their associated risk factors are not consistent and tend to vary across different dairy cattle populations. This variation is attributed to differences in genetics, environmental conditions, farm capacity, morbidity, biosecurity considerations, and farm management practices [4,8,19]. The current study focused on developing knowledge on culling reasons and the related risk factors pertaining to South African Holstein and Jersey cattle herds.

The average length of productive life (739.33 and 696.81 days for Holstein and Jersey, respectively) and the average number of completed lactations (2.37 and 2.47 for Holstein and Jersey, respectively) observed in the current study are comparable to those reported previously for Holstein cows in Italy (2.3 lactations) [25] and high producing Holstein cows in Germany (2.5–3.0 lactations) [26]. Earlier research in South Africa, however, recorded a higher average number of completed lactations for Holstein (2.9) and Jersey (3.1) cows [27]. This may suggest that cow longevity has declined in these South African cattle populations over time, thus posing a risk to the long-term sustainability of the country’s dairy industry. Shorter cow longevity negatively affects the genetic progress, which in turn influence the economic efficiency of milk production, leading to lower herd profitability and poor sustainability. Due to this, factors such as genetic deterioration in fitness and functional traits, increased environmental challenges overtime associated with climate change, and changes in management practices may cause a reduction in longevity [28,29,30,31]. These findings, therefore, highlight the need to pay particular focus and attention on fitness and functional traits in South African dairy cattle as well as implementing management strategies aimed at improving resilience of cows to the environment.

Cow survival rate declined sharply during the early days of productive life and within the early parities for both Holstein and Jersey cows in the current study. Similar results were reported by [32] on Simmental cows in Slovakia. Other studies elsewhere also observed high culling rates during the first lactation, with a gradual decline in culling risk as cows advance in parity [33,34]. In contrast with our findings, other studies showed 40% of Holsteins surviving to 48 months in Argentina [35] and 56% reaching the fourth lactation in Italy [36]. The percentage of cows surviving to a specific length of productive life varies across studies, which may be attributable to differences in the actual culling rates. Besides management factors, breed genotype attributes such as adaptability and resilience to the environment contribute to variation in survival rates of dairy cows during early productive life among different populations. Thus, early lactation management strategies that optimize health and reproduction, as well as breeding of more adapted and resilient cows, may help to improve cow longevity. The major reasons for culling South African Holstein and Jersey cows were infertility, mastitis, and low milk yield. This is concurrence with several previous studies elsewhere [2,6,37,38,39,40,41,42]. However, the relative importance of these culling reasons varies from one study to another. Infertility was markedly the most prevalent culling reason for both Holstein and Jersey, contributing nearly 40% to the total number of Holstein cows that were disposed from the studied herds. This is in line with the widely reported observation that fertility issues are the predominant reason for culling and a significant constraint to dairy production globally [13]. Lower percentages of cows culled due to infertility have, however, been found in Egypt (18.7%) [41] and Canada (23.02%) [42] as well as in pasture-based herds in the Eastern Cape Province of South Africa (7.9%) [13]. The importance of attending to fertility problems in South African dairy herds has been highlighted by previous research, which noted a deterioration in cow fertility traits in populations [43,44]. Increased emphasis on genetic improvement of reproductive traits, as well as implementation of adequate nutritional management and control measures for infectious reproductive diseases, can assist in effectively addressing this problem [45,46].

Mastitis was the second common cause for involuntary culling, accounting for 18.15% and 18.16% of Holstein and Jersey cows culled, respectively. This is comparable to figures obtained from dairy herds in the Eastern Cape (15.1%) [13] and Gauteng (20%) [47], South Africa. Lower incidence rates of culling for mastitis have, however, been reported in Spain (13.5%), New Zealand (4%), and Canada (10.67%) [8,38,41], while higher prevalence was observed in the Free State Province of South Africa (33%) [48] and Egypt (24%) [40]. The incidence of mastitis in South African herds may be attributed to management-related problems such as poor milking hygiene, overcrowding, and increased occurrence of antibiotic resistance, which may also contribute to chronic mastitis infections, particularly in older cows [48]. In addition, production systems used across the herds were found to influence the risk of mastitis, with higher rates observed in pasture-based systems compared to total mixed ration (TMR) systems [47]. Udder problems were the third most prevalent cause of involuntary culling in the Jersey, accounting for 10.89% of cow disposals. This compares well with the prevalence of 9.1% reported for Holstein cows in Iran [49]. Udder problems can lead to high risk of mastitis in cows. Selection for better udder conformation has been effective in reducing the incidence of these issues in many populations [50,51,52]. In Holstein, health issues (11.84%) were the third most important culling reason; however, a much higher rate of 38.6% was reported for Holstein cows in Morrocco [18]. Redwater (33.8%), milk fever (23.3%), and heartwater (6.8%) were found in an earlier study [13] to be the common health problems mainly responsible for dairy cow mortality in the Eastern Cape Province of South Africa. Metabolic and digestive disorders, as well as lameness, were also reported elsewhere as health problems that could contribute to culling dairy cows during early and late lactation [6,53]. Strategic nutritional management of cows during the transitional period is key to minimizing these disorders [6,54]. Similarly to cow fertility, previous research has also noted a declining trend in udder health in the South African dairy cattle population [49]. Thus, there is a need to intensify genetic improvement for resistance to mastitis as well as ensure that sound udder health management practices are followed in South African dairy herds.

Feet and leg problems (5,94%) were the least important reasons for culling Holstein cows, being marginally lower than for Jersey (6.63%) and closely resembling rates of 5.9% and 6.36% recorded in Sweden and Iran, respectively [53,55]. A much lower prevalence of 0.3% was, however, obtained for pasture herds in the Eastern Cape, South Africa [13]. Cows in pasture-based systems, however, typically have a lower occurrence of foot and leg problems compared to those in confined housing systems due to the reduced exposure to concrete and manure, which are known risk factors for hoof problems [56,57]. A low incidence of culling due to feet and leg problems may also reflect effective hoof health and lameness prevention programs. Injury (3.64%) was the least common reason for culling Jersey cows, corresponding to the prevalence of neck (2.0 ± 4.1%) and back injuries (3.6 ± 3.4%) observed in dairy herds in Wisconsin, USA [58].

Low milk yield was the only voluntary culling reason recorded, and it had rates of 11.76% and 19.76% for Holstein and Jersey, respectively. These frequencies are higher than those observed in Morocco (1.2%) [18] and New Zealand (9%) [38] as well as in a local population studied in the Eastern Cape (7.1%) [13] A higher prevalence of 23.4% was, however, reported for high producing dairy herds in Spain [8]. Culling for low milk yield presents an opportunity to improve the herd’s genetic merit for milk production through selection, thereby enhancing overall herd profitability. The greater the proportion of cows available for selection, the higher the selection pressure and resultant rate of genetic gain. This underscores the need to minimize involuntary culling to achieve better opportunities for voluntary culling. The results of the current study suggest that there might be larger scope for genetic improvement in Jersey due to the higher rate of voluntary culling.

Although voluntary culling rates were overall not bad, there was an increase in involuntary culling for both Holstein and Jersey over the study period. Similar findings were made in previous studies in Iran and Morrocco [18,54], suggesting that cow longevity may be declining. Previous research in South Africa recorded a decline in longevity of dairy cows from the year 2003 to 2012, with only small proportions of Holstein (9%) and Jersey (7%) cows reaching the fifth lactation [59], thus supporting this observation. Functional longevity of South African Holstein cows was also found to have decreased between 1995 and 2007 [60], also indicating increased voluntary culling. These trends further underscore the need to pay particular focus and attention on minimizing involuntary culling in South African dairy herds.

Compared to other seasons, summer calving was associated with the highest risk of culling in South African Holstein and Jersey cows. This concurs with previous studies from Sweden, Iran, and Germany [61,62,63] that also reported a higher risk of culling during hotter seasons (summer and spring) than in cooler seasons (autumn and winter). Increased effects of heat stress on the cows, which compromise their health, fertility, and production, may be attributable to these results [5,64]. Summer in South Africa is characterized by high temperatures and humidity. Average temperatures are above 35 °C in most parts of the country, with humidity levels generally exceeding 60%. Such climatic conditions are associated with extreme heat stress, especially if there are no mitigation strategies in place.

Risk of culling increased with parity, peaking in third parity, followed by a drop to the lowest in fourth and later lactation cows. Similar results have been found in several other studies [18,52,62] and may be due to reluctance by farmers to cull cows in early productive life before cows have repaid replacement and rearing costs and generated profit [65,66] or increased health disorders associated with advancing age (parities). By the fourth parity, cows would have demonstrated consistent reproductive and production performance, with reduced incidence of reproductive failure, metabolic disorders, and disease indicating greater health resilience and physiological maturity, leading to a lower risk of culling [67,68].

Cows in small South African herds had the highest risk of being culled, which is contrary to findings from several other studies [6,69,70,71]. Large herd size has widely been associated with the highest risk of culling mainly due to difficulties in maintaining effective biosecurity and providing adequate housing facilities, which increases the risk of infectious diseases [72,73,74]. High culling rates, indicating elevated risk, have been observed previously in shrinking South African Holstein herds [59]. Small herds in South Africa are mostly characterized by low levels of inputs such as feed, health care, and housing infrastructure, as well as inadequate management practices, resulting in low productive and reproductive performance and poor udder health of cows [75]. These conditions may predispose cows to a higher risk of being removed from the herd prematurely.

The outcomes of this study suggest that strategic management practices can be developed with major focus to the improvement of fitness and functional traits to reduce economic losses associated with involuntary culling, enhancing cow resilience and ensuring profitable as well as sustainable dairy farming. Although these findings provide valuable insights into the status of culling in South African dairy herds, and the results can be gainfully applied, this study was somehow limited by the quality of the culling records. Data were recorded by farmers by assigning a lactation termination or disposal reason obtained from a prescribed list to each lactation record. The farmers were, however, not provided with comprehensive guidelines or clear definitions of the culling reasons. Some culling reasons, such as poor temperament, are also not available on the list of disposal reasons. Furthermore, the recording system only has provision for recording one culling reason. A cow might, however, have numerous reasons to warrant its removal from the herd, which means that sometimes the recorded culling reason is only one of many possibilities. Thus, the recorded reason may not reflect the primary cause for removing the cow from the herd. Quality and utility of the data could be improved by documenting destination of all cows leaving the herd and allowing for the recording of one or multiple reasons for herd exit [76]. More value could also be derived from the culling data if culling reasons were made more specific; for example, indicating the disease instead of just recording health issues.

## 5. Conclusions

This study found the average length of productive life for South African Holstein and Jersey cows to be between two and three lactations, with large-scale culling taking place during early productive life. This suggests low resilience by cows, which may pose a threat to long-term profitable and sustainable dairy production. Most cows are removed from their herds involuntarily due to infertility, mastitis, udder problems, and health issues, thus highlighting the need to pay particular focus on improving fitness and functional traits in the population. This can be achieved by ensuring that these traits receive adequate emphasis in the breeding objectives, as well as increasing their accuracy of selection through genomic techniques. The observed increase in rates of involuntary culling over time further underscores the importance of paying due attention to cow resilience in this population. Implementing herd management practices that optimize cow reproduction and health may also assist in addressing this problem. Such practices may include providing cooling facilities during hot months, as summer calving cows were found to increase the odds for culling, presumably due to the negative impacts of heat stress on reproduction and health. Paying particular focus and attention on high-culling risk groups, such as third parity cows, may also contribute towards minimizing involuntary culling in these herds. Future research may focus on application of techniques like genomic selection to accelerate genetic improvement of functional traits such as longevity, fertility, and health, since they have low heritability. In addition, comprehensive analysis on factors influencing the proportions of voluntary and involuntary culling is critical for the improvement of herd performance.

## Figures and Tables

**Table 1:** Restrictions for age of cow at calving by lactation number.

Lactation Number	Calving Age (Months)	
	Holstein	Jersey
1	20–42	17–40
2	30–54	29–53
3	40–67	41–67
4	50–79	53–77
5	60–91	65–89
6	70–103	77–101
7	80–115	89–113
8	90–127	113–137

**Table 2:** Descriptive statistics for length of productive life (days), number of completed lactations, milk yield, and age at first calving for South African Holstein and Jersey cows.

Breed	Trait	Mean	SE	Minimum	Maximum
Holstein	Length of productive life (days)	739.33	434.31	1	2124
	Number of lactations	2.37	1.08	1	7
	Age at first calving (months)	28.6	7.9	20.0	143.0
	Milk yield in (kg)	8912	4927	595	47,337
Jersey	Length of productive life (days)	696.81	415.44	1	2121
	Number of lactations	2.47	1.13	1	8
	Age at first calving (months)	26.9	6.7	17.0	139.0
	Milk yield in (kg)	5150	2466	483	24,569

SE = standard error; kg = kilograms.

**Table 3:** Summary statistics for factors associated with the odds of culling South African Holstein and Jersey cows.

Factor	Holstein			Jersey		
	Wald-*χ*^2^	SE	*p*-Value	Wald-*χ*^2^	SE	*p*-Value
Calving season	48.04	0.024	<0.0001	64.47	0.031	<0.0001
AFC	0.04	0.023	0.8234	1.10	0.026	0.2939
Parity	10.41	0.011	0.0013	3.88	0.013	0.0486
Herd size	128.80	0.014	<0.0001	107.86	0.013	<0.0001

AFC = age at first calving; SE = standard error.

**Table 4:** Estimated odds ratio, 95% Wald confidence interval (CI), and *p*-value for culling in South African Holstein and Jersey cows in different season, parity, and herd size groups.

Breed	Factor	Odds Ratio	95% Wald Confidence Intervals		*p*-Value
			Lower	Upper	
Holstein	**Calving season**				
	Summer	1.000	-	-	-
	Winter	0.752	0.703	0.804	**<0.0001**
	Autumn	0.829	0.775	0.888	**0.174**
	Spring	0.850	0.792	0.913	0.868
	**Parity**				
	1	1.000	-	-	-
	2	1.205	1.127	1.288	**<0.0001**
	3	1.461	1.364	1.564	**<0.0001**
	4	0.855	0.797	0.916	**<0.0001**
	**Herd size**				
	Small	1.000	-	-	-
	Small–medium	0.898	0.843	0.957	**<0.0001**
	Medium	0.842	0.789	0.900	**<0.0001**
	Medium–large	0.487	0.423	0.560	**<0.0001**
	Large	0.659	0.611	0.712	**<0.0001**
Jersey	**Calving season**				
	Summer	1.000	-	-	-
	Winter	0.671	0.616	0.731	**<0.0001**
	Autumn	0.847	0.773	0.927	0.797
	Spring	0.879	0.805	0.959	0.097
	**Parity**				
	1	1.000	-	-	-
	2	1.170	1.070	1.280	0.076
	3	1.465	1.341	1.602	**<0.0001**
	4	0.897	0.825	0.979	**<0.0001**
	**Herd size**				
	Small	1.000	-	-	-
	Small–medium	0.879	0.808	0.956	**<0.0001**
	Medium	0.446	0.404	0.494	**<0.0001**
	Medium–large	0.740	0.660	0.829	0.619
	Large	0.854	0.774	0.943	**0.002**

## Data Availability

The study’s original findings are fully outlined in this article. Any additional details are available on request from the corresponding author.
